# Nimotuzumab Combined With Irradiation Enhances the Inhibition to the HPV16 E6-Promoted Growth of Cervical Squamous Cell Carcinoma

**DOI:** 10.3389/fonc.2020.01327

**Published:** 2020-08-05

**Authors:** Zhonghua Xu, Hang Shu, Fan Zhang, Weiwei Luo, Yan Li, Jinjin Chu, Qihong Zhao, Yin Lv

**Affiliations:** ^1^Department of Radiotherapy, The First Affiliated Hospital of Anhui Medical University, Hefei, China; ^2^Department of Nutrition and Food Hygiene, School of Public Health, Anhui Medical University, Hefei, China

**Keywords:** cervical squamous cell carcinoma, human papillomavirus, epidermal growth factor receptor, nimotuzumab, radiosensitivity

## Abstract

Human papillomavirus (HPV) 16 E6 has been proved to increase the radiosensitivity and lead to the EGFR overexpression in cervical cancer cells. In this study, to investigate the inhibition of nimotuzumab-mediated EGFR blockade combined with radiotherapy, we established a C33A cervical squamous cell line overexpressed HPV16-E6 and a nude mouse model bearing these cell lines. The CCK-8 assay was used to detect the effects of various treatments on the proliferation of C33A cells. Flow cytometry was used to detect the rates of apoptosis and cell cycle arrest. Gene transcription and protein expression were detected by quantitative real-time polymerase chain reaction (qRT-PCR) and western blot, respectively. Immunohistochemical staining was used to evaluate protein expression in tumor tissue. We revealed that E6-overexpressing C33A cells grew faster and were more sensitive to radiotherapy than control cells *in vitro* and *in viv*o. The expression levels of EGFR, as well as those of downstream signaling molecules AKT and ERK 1/2, were significantly upregulated in C33A cells that overexpressed E6. We observed that nimotuzumab combined with radiotherapy could enhance the inhibition of C33A cell growth induced by E6, both *in vitro* and *in vivo*. We also observed enhanced effect after combination on G2/M cell cycle arrest and apoptosis in E6-overexpressing C33A cells. Furthermore, the combined therapy of nimotuzumab and radiation remarkably reduced the protein expression levels of EGFR, AKT, ERK 1/2 *in vitro*, and *in vivo*. In conclusion, HPV16 E6 expression is positively correlated with levels of EGFR, AKT, and ERK 1/2 protein expression. The combined treatment with nimotuzumab and radiotherapy to enhance radiosensitivity in E6-positive cervical squamous cell carcinoma was related to enhanced G2/M cell cycle arrest and caspase-related apoptosis.

## Introduction

Cervical cancer is the second most common malignant tumor and the fourth major cause of death among women worldwide. Cervical squamous cell carcinoma (CSCC) accounts for 90% of cervical cancer (1). The WHO estimates that there were 570,000 new cases in 2018, accounting for 6.6% of the total number of female cancers and representing a serious threat to women's health (2). Over the past 20 years, most patients with locally advanced cervical cancer (LACC) have been treated with concurrent chemoradiotherapy (CCRT) regimens that include concurrent treatment with radiotherapy and platinum-based treatment. The results of five randomized controlled clinical trials conducted by the Radiation Therapy Oncology Group (RTOG), the Gynecological Oncology Group (GOG), and the Southwest Cancer Group (SWOG) demonstrated that CCRT can significantly improve overall survival (OS) and progression-free survival (PFS) in patients with cervical cancer (3), making CCRT the “gold-standard” treatment for LACC. Although the 5-year survival rate of CCRT is 16% higher than that of radiotherapy alone, the treatment responses are not satisfactory, and about 50% of patients have local recurrence and distant metastasis within 2 years of the initial CCRT session (4). Specific survival data showed that the 5-year survival rate among stage III patients who receive CCRT is about 30%, while the 5-year survival rate of stage IV patients who receive CCRT is <15% (5, 6). Therefore, efforts to clarify the radiosensitivity of a given tumor type and to identify factors that contribute to insensitivity are very important for improving curative effects and prolonging survival in patients with LACC.

Human papillomavirus (HPV) is a non-enveloped DNA virus that infects human epithelial tissues. Infection with a high-risk HPV genotype is considered one of the most important causes of cervical cancer. HPV18 causes about 15% of cases; HPV16 causes about 55% of cervical cancer cases; HPV16 infection is mainly related to the occurrence of cervical squamous cell carcinoma (7, 8). In 1985, Seedorf et al. (9) published the DNA genomic sequence of HPV16. Analyses of HPV16 expression products revealed that the HPV16 genome contains six early open reading frames (ORF), including E1, E2, E4, E5, E6, and E7. To investigate which ORF plays a major role in the oncogenesis of HPV16, Yutsudo et al. (10) constructed recombinant mouse retrovirus containing various DNA fragments from the HPV16 genome and found that E6 and E7 are HPV16-transforming genes. E6 and E7 ORFs can act independently to induce malignant transformation. These two proteins are sufficient to maintain transformation status in cervical cancer cell lines (11). In addition, E6 and E7 were found to be continuously expressed in HPV-infected cervical cancer tissues (12). Additional studies demonstrated that E6 and E7 bind to tumor suppressor genes P53 and Rb, respectively, resulting in rapid degradation of P53 and the loss of Rb products. E6 and E7 thus appear to contribute to the occurrence and development of cervical cancer in different ways (13). Interestingly, HPV16 E6 can also induce telomerase activity (14). In addition, a unique feature of HPV16 E6 oncoprotein is the presence of a PDZ, namely the proteins of PSD-95 (post-synaptic density-95 kDa; also SAP90), DLG (Drosophila major disc), and ZO-1 (zonula occludens 1), binding motif (PBM) at the C-terminus. Through this motif, E6 can interact with proteins containing PDZ domains. The HPV16 E6 protein has multiple cell-binding partners, including the human homologs of DLG (hDLG), Scribble, MUPP1 (multi-PDZ protein-1), MAGI (membrane-associated guanylate kinase inverted)-1, MAGI-2, and MAGI-36 (15). Importantly, deletion analysis revealed that the PDZ domain of HPV16 E6 is not involved in p53 binding and degradation (16). So, focused on the function of HPV16 E6 could give us more hints to understand its other variants. Although, at present, the HPV vaccine is a viable means to reduce the incidence of cervical cancer, it will take decades to observe the overall effect in terms of public health. To further improve the effectiveness of treatment for HPV16-positive cervical cancer, novel strategies are needed to target the special molecular pathological status of HPV infections.

Several host proteins other than P53 have been found to play a role in oncogenesis related to E6. In 1995, Peto et al. (17) reported an interaction between epidermal growth factor receptor (EGFR) signaling and HPV16 oncogene expression. It has been known that cervical cancer with EGFR overexpression is associated with poor therapeutic efficacy and prognosis. HPV infection and the EGFR pathway have been identified as targets for cervical cancer therapy. Nimotuzumab is a humanized IgG1 isotype monoclonal antibody used in the treatment of malignant tumors (18, 19). In the present study, to investigate the combined effect of nimotuzumab-mediated EGFR blockade and radiotherapy on E6-promoted cervical cancer growth, we established an HPV-E6–overexpressing C33A cervical squamous cell line and a nude mouse model bearing these cells. We also explored the correlation between HPV6 E6, EGFR, and radiotherapy by treating one group with nimotuzumab plus radiotherapy and one group with radiotherapy alone. Possible effects on apoptosis and cell cycle regulation were also investigated.

## Materials and Methods

### Cell Culture and Transduction

The HPV-negative human cervical squamous cell line C33A, which carries p53 mutations, was obtained from the Cell Resource Center, Shanghai Institute of Biochemistry and Cell Biology at the Chinese Academy of Sciences (Shanghai, China). C33A and the derived cells were cultured at appropriate density with DMEM supplemented with 10% (v/v) fetal bovine serum (FBS) and 1% antibiotics (100 μg/mL streptomycin and 100 U/mL penicillin) in a humidified incubator under 5% CO_2_ conditions, at 37°C. The recombinant lentivirus vector carrying the E6 overexpression cassette was constructed, and transduction was performed as described previously (20). Gene-overexpressing cell lines were established with puromycin screening and confirmed by quantitative real-time polymerase chain reaction (qRT-PCR) and western blot.

### Real-Time PCR

Total RNA was isolated from whole-cell lysate with TRIzol, according to the manufacturer's instructions (Invitrogen, Thermo Fisher). RNA was reverse-transcribed, and the synthesized cDNA was subjected to qPCR using the PrimeScript RT reagent kit (TaKaRaBio, Beijing, China), according to the manufacturer's instructions. Glyceraldehyde 3-phosphate dehydrogenase (GAPDH) was used as the internal control for normalizing the expression of target genes. The specific primers used are listed in Table 1. The melting curves and *E* = 2^−ΔΔCt^ algorithm were analyzed with LightCycler software (Roche Diagnostics).

**Table 1:** Primers for RT-PCR.

**Genes**	**Sequences**	**Size (bp)**	**GenBank Accession**
HPV16 E6	FORWARD: CAGGAGCGACCCAGAAAGTT	206	NM.152686.4
	REVERSE: CTGTTGCTTGCAGTACACACA		
EGFR	FORWARD: ACCCATATGTACCATCGATGTC	97	NM.001346897.2
	REVERSE: GAATTCGATGATCAACTCACGG		
ATK	FORWARD: TGACCATGAACGAGTTTGAGTA	110	NM.001382431.1
	REVERSE: GAGGATCTTCATGGCGTAGTAG		
ERK1/2	FORWARD: ATGGTGTGCTCTGCTTATGATA	188	NM.002745.5
	REVERSE: TCTTTCATTTGCTCGATGGTTG		
GAPDH	FORWARD: AATGAAGGGGTCATTGATGG	108	NM.001357943.2
	REVERSE: AAGGTGAAGGTCGGAGTCAA		

### Western Blot

Total protein was extracted from cell lysate with RIPA lysis buffer (Beyotime Biotechnology, Shanghai, China). The protein concentration was determined using a BCA protein concentration quantification kit (Beyotime Biotechnology, Shanghai, China). Denatured proteins were separated by SDS-PAGE and transferred to PVDF membranes using the X cell Surelock system (Thermo Fisher Scientific, Shanghai, China). Membranes were blocked with 5% non-fat dry milk in Tris-buffered saline with 0.05% Tween 20 (TBST) and probed with primary antibodies overnight at 4°C. The primary antibodies used included HPV16 E6 (cat. no. ab70, 1:1,000 dilution, Abcam, USA), EGFR (cat. no. 4267S, 1:1,000 dilution, Cell Signaling Technology, USA), Akt (cat. no. 4685S, 1:1,000 dilution, Cell Signaling Technology, USA), ERK1/2 (cat. no. 9102S, 1:1,000 dilution, Cell Signaling Technology dilution, USA), Fas (cat. no. 4233S, 1:1,000 dilution, Cell Signaling Technology, USA), caspase-3 (cat. no. ab197202, 1,000 dilution, Abcam, USA), and GAPDH (cat. no. 5174S, 1:1,000 dilution, Cell Signaling Technology, USA). After washing, membranes were incubated with goat HRP-conjugated secondary antibodies at room temperature for 2 h (1:50,000 dilution, Boster Biological Technology Co., Ltd., Wuhan, China). The reactions were detected with an ECL western blot detection kit (Pierce, Waltham, MA, USA). The detected bands were visualized and semi-quantified with a Gel Image System manufactured by Tanon Fine Do X6 (Tanon Science and Technology Co., Ltd., China).

### Cell Proliferation Assay

Cell proliferation rates were determined with the CCK8 assay (Beyotime Biotechnology, Shanghai, China), according to the manufacturer's instructions. Six replicates were tested for each group. Specifically, cells at a density of 4 × 10^4^ mL^−1^ were seeded into 96-well-plates for 16 h, then serum-starved overnight. In treatment group, after the medium was replaced with complete medium containing 400 μg/mL nimotuzumab and incubated for 2 h, cells received 1.8 Gy X-ray irradiation for 24, 48, or 72 h, which the concentration of nimotuzumab and the dosage of X-ray irradiation were applied from the previous studies (21–23). After each well had been incubated with 10 μL CCK-8 reagent for 2 h, cells were counted by measuring absorbance at 450 nm.

### Cytometry

Cells were harvested and stained with propidium iodide (PI) after pretreatment with RNase A for cell-cycle analysis. The Annexin-AbflourTM647 Apoptosis Detection Kit (Abbkine Scientific, Beijing, China) was used for detecting cell apoptosis, according to the manufacturer's instructions. The FACS Canto II instrument (BD Biosciences, San Diego, CA, USA) was used to perform cytometry experiments. FlowJo software (Version 10.0.7) was used to analyze progression through the cell cycle and apoptosis.

### Tumor-Bearing Mouse Model

Four-to-six-week-old female BALB/C nude mice weighing 18–22 g were obtained from the Beijing Vital River Laboratory Animal Technology Co., Ltd., (Beijing, China). Mice were bred and housed under specific pathogen-free (SPF) conditions at the laboratory animal center of Hefei Institutes of Physical Science, Chinese Academy of Sciences. C33AE6 cells were pretreated with 400 μg/mL nimotuzumab and/or 1.8 Gy of 6MV X-ray for 2 h. To establish the implanted tumor model, 1 × 10^6^ cells suspended in 100 μl PBS mixed with Matrigel at a ratio of 1:1 were injected subcutaneously into the dorsal sides of randomly selected nude mice (*n* = 5 per group). The Animal Care and Use Committee at Anhui Medical University approved all experimental procedures, which were designed to minimize animal suffering.

### Measurement of Body Weight and Tumor Size

After the hard nodules appeared at the site of inoculation 7–10 days post-implantation, tumor size was measured every 3 days. Tumor volume (mm^3^) was measured with Vernier calipers, with the following formula: tumor volume = 1/2ab^2^ [a, long diameter (mm); b, transverse diameter (mm)]. Mouse weight and tumor size were recorded in detail to establish a tumor growth curve. On day 28, mice were sacrificed by cervical dislocation after chloral hydrate anesthesia. The tumors were removed, photographed, and measured.

### Immunohistochemical (IHC) Staining

Briefly, mouse xenograft tumor samples fixed in 4% buffered paraformaldehyde were dehydrated in an ethanol series, cleared in xylene, and embedded in paraffin. Paraffin-embedded tissues were sectioned into 4 μm-thick slices using a rotary microtome. After pretreatment with 3% hydrogen peroxide at room temperature for 5–10 min, the slides were placed in antigen repair solution, and then incubated in a microwave. After blocking in goat serum, sections were incubated with primary antibody against caspase 3 (1:1,000) and cleaved-caspase 3 (1:200) (cat. no. 9664S, Cell Signaling Technology, USA) overnight at 4°C. Slides were washed and incubated with biotinylated secondary antibody and horseradish-labeled streptavidin working solution (ZSGB-BIO, China) at room temperature for 2 h. Stained tissues were developed in DAB colorant (ZSGB-BIO, China), then counterstained with hematoxylin. The results of IHC staining were independently evaluated by two pathologists and quantified according to the degree of staining, as follows: 0 (no staining), 1 (light yellow), 2 (light brown), and 3 (brown). Slides were graded according to the number of positive cells as follows: 0 (<0%), 1 (<30%), 2 (30–60%), and 3 (>60%). The two scores were evaluated as follows: 0–1 (−), 2 (+), 3–4 (+ +), ≥5 (+ + +). Finally, (−, +) was defined as low expression, and (+ +, + + +) was defined as high expression.

### Statistical Analysis

Data were analyzed with SPSS 23.0 (IBM Corp, Armonk, NY, USA) and GraphPad Prism 8.0 software (GraphPad Software Inc., CA, USA). For data that were normally distributed, results are presented as mean ± standard deviation. One-way ANOVA was used for comparing multiple groups, and Students' *t*-test was used for pairwise comparisons. *P* < 0.05 was set to indicate a significant difference.

## Results

### HPV16 E6 May Enhance CSCC Cell Proliferation via the Upregulation of EGFR

**Figure 1:**
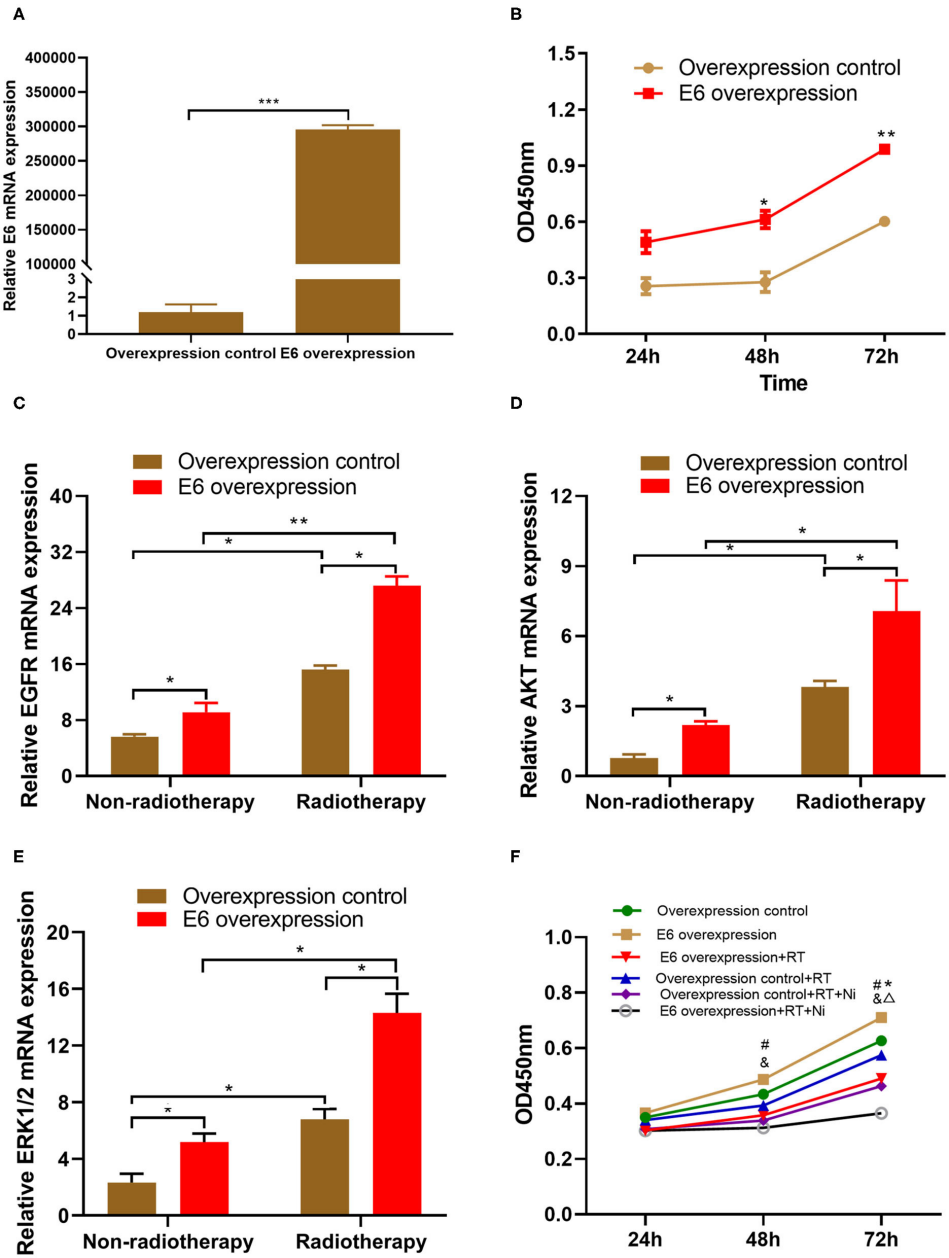
**(A)** Quantitative RT-PCR demonstrated that the expression level of HPV16 E6 was significantly higher in the E6-overexpressing C33A cell line, C33AE6, than in the vehicle-transduced C33A cell line, C33AT (***P < 0.001). **(B)** The results of a CCK-8 assay showed that the OD450 for C33AE6 became significantly higher than that of C33AT at 48 h post-seeding (*P < 0.05; **P < 0.01). **(C–E)** qRT-PCR results showed that the expression levels of EGFR, AKT, and ERK1/2 were significantly higher in C33AE6 cells than in C33AT cells (*P < 0.05; **P < 0.01). **(F)** The CCK-8 assay revealed that the OD450 value was significantly lower in C33AE6 cells treated with a single dose of irradiation (RT) or irradiation in combination with nimotuzumab (RT+Ni), compared with untreated C33AE6 cells. This effect was first observed at 72 h post-treatment and then persisted throughout the study period (*^&^*P* < 0.05). OD450 values were significantly lower in C33AE6 cells that received RT+Ni treatment, compared with C33AE6 cells treated with RT alone. This effect was first observed at 72 h post-treatment and then persisted throughout the study period (^Δ^*P* < 0.05). (^#^*P* < 0.05, Overexpression control vs. E6 overexpression; *P < 0.05, RT group vs. Control group; ^&^*P* < 0.05, RT+Ni group vs. Control group; ^Δ^*P* < 0.05, RT+Ni group vs. RT group).

As shown in Figure 1A, the results of qRT-PCR demonstrated that the expression level of HPV16 E6 was significantly higher in E6-overexpressing C33A cells (C33AE6), compared with vehicle-transduced C33A cells (C33AT) (*P* < 0.05). There was no significant difference in E6 expression levels between C33A and C33AT cells. These results indicate that stable overexpression of E6 was established in C33AE6 cells. As shown in Figure 1B, the CCK-8 assay results revealed that the OD_450_ of C33AE6 cells was greater than that of C33AT cells from 48 h post-seeding onward (*P* < 0.05), indicating that the overexpression of E6 enhanced C33A cell growth. The results of RT-PCR (Figures 1C–E) showed that the expression levels of EGFR, AKT, and ERK 1/2 were significantly higher in C33AE6 cells than in C33AT cells (*P* < 0.05), indicating that the expression of EGFR and downstream molecules AKT and ERK 1/2 were upregulated by the overexpression of E6. Taken together, these data suggest that E6 may enhance the proliferation of C33A cells via activation of the EGFR signaling pathway, which can be inhibited by EGFR blockade.

### Nimotuzumab Combined With Radiotherapy Enhances the Inhibition to the E6-Promoted Growth in C33A Cells

We conducted several experiments to determine whether EGFR blockade could enhance the irradiation-mediated inhibition of cervical cell growth associated with E6 expression. As shown in Figure 1F, the OD_450_ values of pretreated C33AE6 cells treated with a single session of irradiation (RT) or with irradiation in combination with nimotuzumab (RT+Ni) were significantly lower than the OD_450_ values of untreated C33AE6 cells, from 72 h post-treatment onward (*P* < 0.05). In addition, the OD_450_ value of C33AE6 cells that received RT+Ni treatment was also significantly lower than that of C33AE6 cells treated with RT alone; this difference was detectable from 72 h post-treatment onward (*P* < 0.05). These findings indicate that nimotuzumab combined with radiotherapy may enhance the inhibition to E6-promoted C33A cell growth *in vitro*.

### Nimotuzumab Combined With Radiotherapy Increase Apoptosis and G2/M Cell Cycle Arrest in E6-Overexpressing CSCC

**Figure 2:**
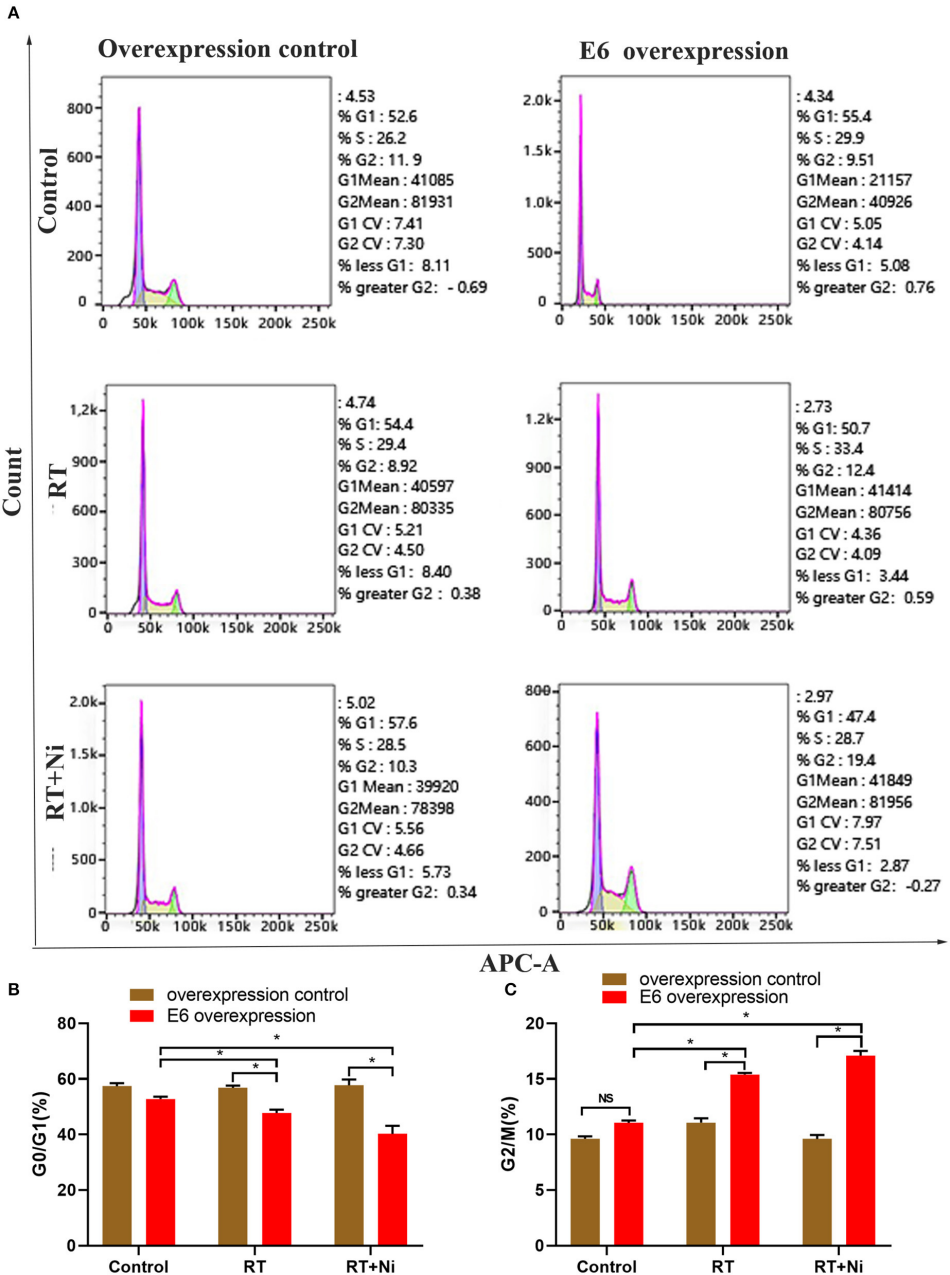
**(A–C)** The proportion of G0/G1 cells among RT+Ni-treated C33AE6 cells was significantly lower than that among untreated cells (*P < 0.05). The proportion of G2/M C33AE6 cells in the RT+Ni treatment group was higher than that in the control group (*P < 0.05).

**Figure 3:**
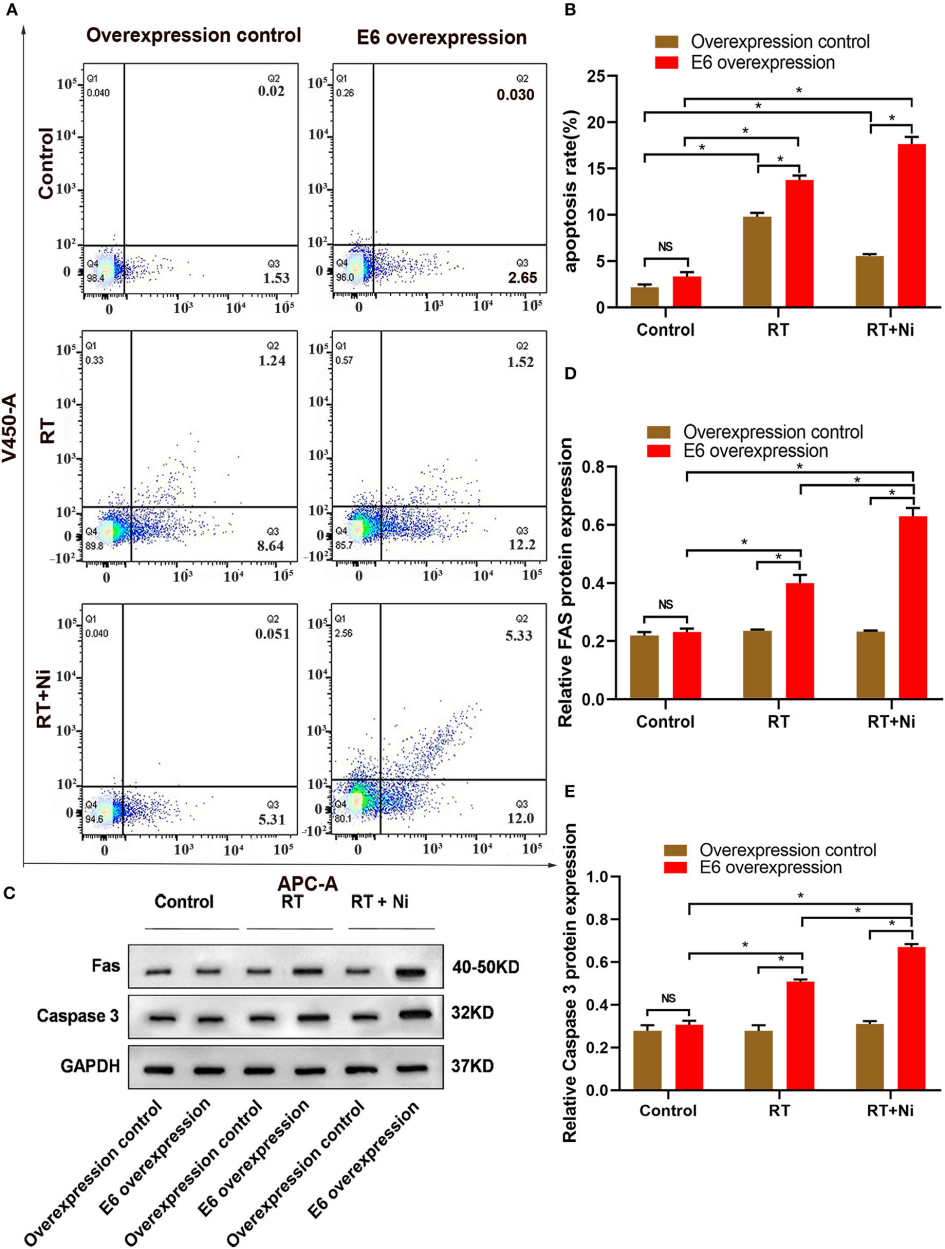
**(A,B)** Flow cytometry results show that the apoptosis rate of C33AE6 cells was significantly higher in the RT group and the RT+Ni combination group, compared with untreated cells (*P < 0.05). The apoptosis rate was also significantly higher in the RT+Ni group than in the RT group (*P < 0.05). **(C–E)** Expression levels of Fas and caspase 3 in C33AE6 cells were measured by western blot. The results showed significantly higher levels in the RT group and the RT+Ni group, compared with untreated cells (*P < 0.05). The expression levels of Fas and Caspase 3 were significantly higher in the RT+Ni group of C33AE6 cells, compared with the RT group (*P < 0.05).

As shown in Figures 2A–C, the proportion of RT+Ni-treated C33AE6 cells in the G0/G1 phase was significantly lower than that of untreated cells (*P* < 0.05). The proportion of G2/M C33AE6 cells in the RT+Ni treatment group was higher than that in the control group, and this difference was statistically significant (*P* < 0.05). As shown in Figures 3A,B, the results of flow cytometry experiments showed that the apoptosis rate of C33AE6 cells was significantly higher in the RT group and the RT+Ni combination group, compared with untreated cells (*P* < 0.05). In addition, the rate of apoptosis in the RT+Ni group was significantly higher than in the RT group (*P* < 0.05; Figures 3A,B). We also performed western blot to measure protein levels of apoptosis markers. As shown in Figures 3C–E, the expression levels of Fas and caspase 3 in C33AE6 cells were significantly higher in the RT group and the RT+Ni group, compared with untreated cells (*P* < 0.05). The expression levels of Fas and Caspase 3 in RT+Ni C33AE6 cells were significantly higher than in the RT C33AE6 cells (*P* < 0.05). Collectively, these results suggest that treatment-induced G2/M arrest and apoptosis. Thus, administering nimotuzumab in combination with radiotherapy inhibited C33AE6 cell growth.

### Nimotuzumab Combined With Radiotherapy Can Significantly Inhibit the Activation of EGFR, AKT, and ERK 1/2 Signaling in C33A Cells

**Figure 4:**
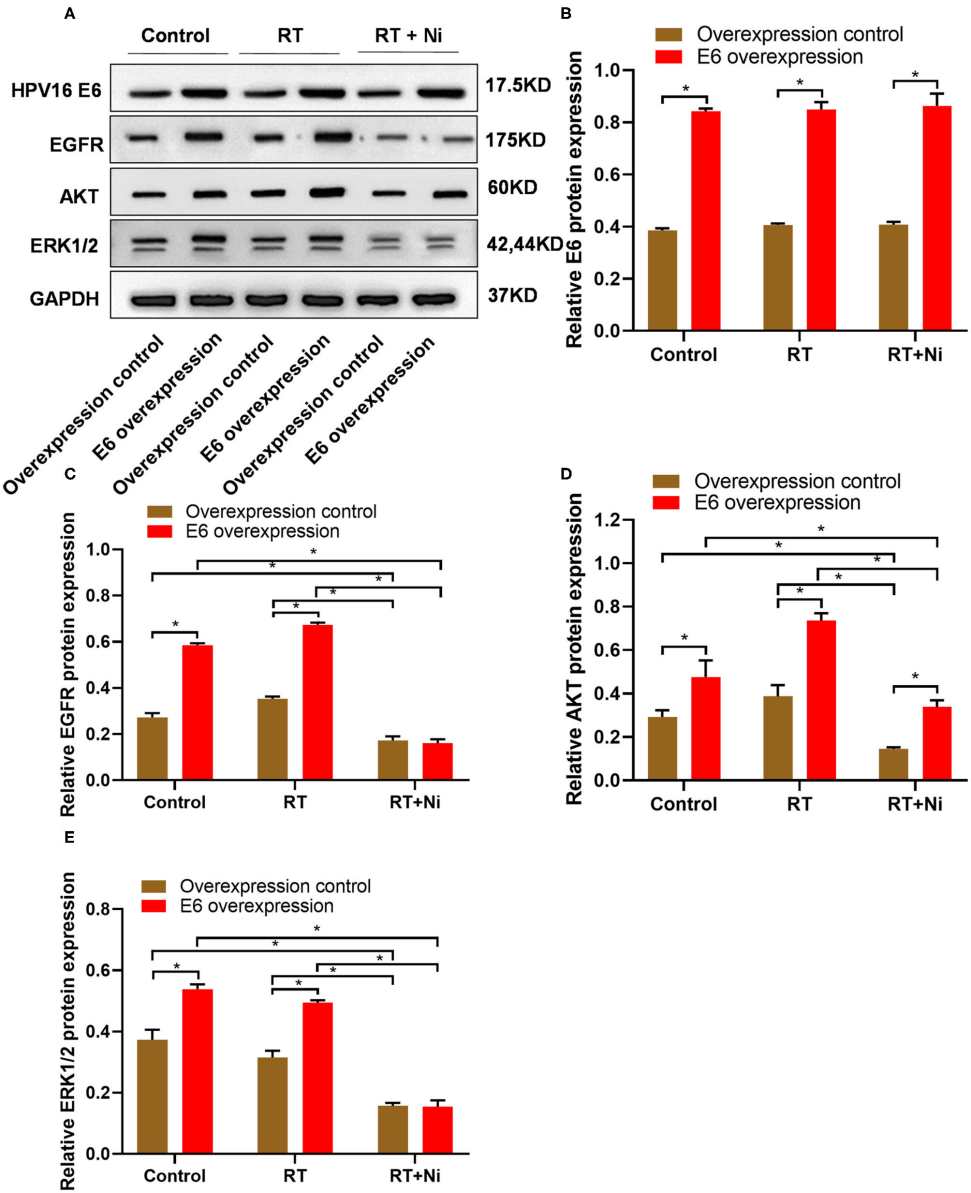
**(A–E)** Western blot results showed that the expression levels of EGFR, AKT, and ERK1/2 were significantly higher in C33AE6 cells than in C33AT cells (*P < 0.05). In addition, compared with the control group and the radiotherapy group, radiotherapy combined with nimotuzumab significantly reduced the expression levels of EGFR, AKT, and ERK 1/2 (*P < 0.05).

To further elucidate the molecular mechanism of enhanced cytotoxicity of nimotuzumab combined with radiotherapy, we detected the expression levels of E6, EGFR, AKT, and ERK 1/2 in C33A cells by Western blot (Figure 4A). We observed that the expression of E6 was up-regulated in the overexpression groups and had little response to the radiotherapy (RT) or combined therapy (RT+Ni), but significantly increased compared with the overexpression control groups (Figure 4B). Compared with the control group, radiotherapy can slightly increase the expression levels of EGFR and AKT in C33A cells, while the expression levels of ERK 1/2 did not change significantly. In addition, compared with the control group and the radiotherapy group, radiotherapy combined with nimotuzumab significantly reduced the expression levels of EGFR, AKT, and ERK 1/2 (Figures 4C–E, *P* < 0.05). These data indicate that radiotherapy combined with nimotuzumab can significantly inhibit EGFR-related AKT and ERK 1/2 activation in C33A cells.

### Nimotuzumab Combined With Radiotherapy Enhances the Inhibition of E6-Promoted C33A Cell Growth *in vivo*

**Figure 5:**
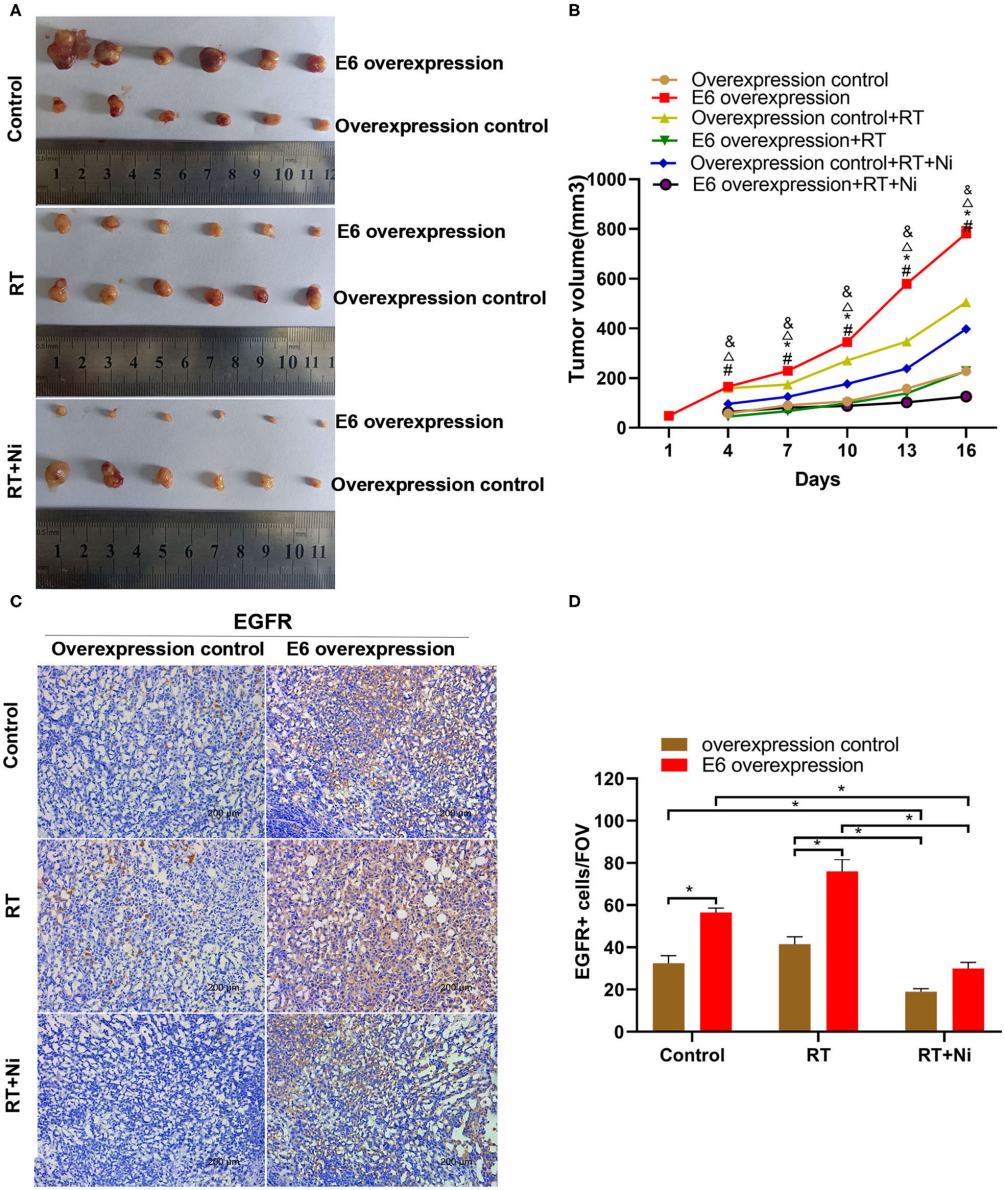
**(A,B)** In nude xenograft mice, the tumor growth of C33AE6 cells was significantly more rapid than that of C33AT cells. This effect was first observed 4 days after injection and then persisted throughout the study period (^#^*P* < 0.05). From 7 days after injection until the end of the study period, tumor growth was significantly slower in the RT and RT+Ni treatment groups, compared with the control group (*^&^*P* < 0.05). Tumor growth in the RT+Ni group was significantly slower than that in the RT group from 7 days post-injection to the end of the study (^Δ^*P* < 0.05). (^#^*P* < 0.05, Overexpression control vs. E6 overexpression; *P < 0.05, RT group vs. Control group; ^&^*P* < 0.05, RT+Ni group vs. Control group; ^Δ^*P* < 0.05, RT+Ni group vs. RT group). **(C,D)** Expression levels of EGFR were slightly higher in the RT group compared with the control group. In addition, compared with the control group and the radiotherapy group, radiotherapy combined with nimotuzumab significantly reduced the expression levels of EGFR (*P < 0.05). Data are representative images (magnification × 200) from each group of tumors.

**Figure 6:**
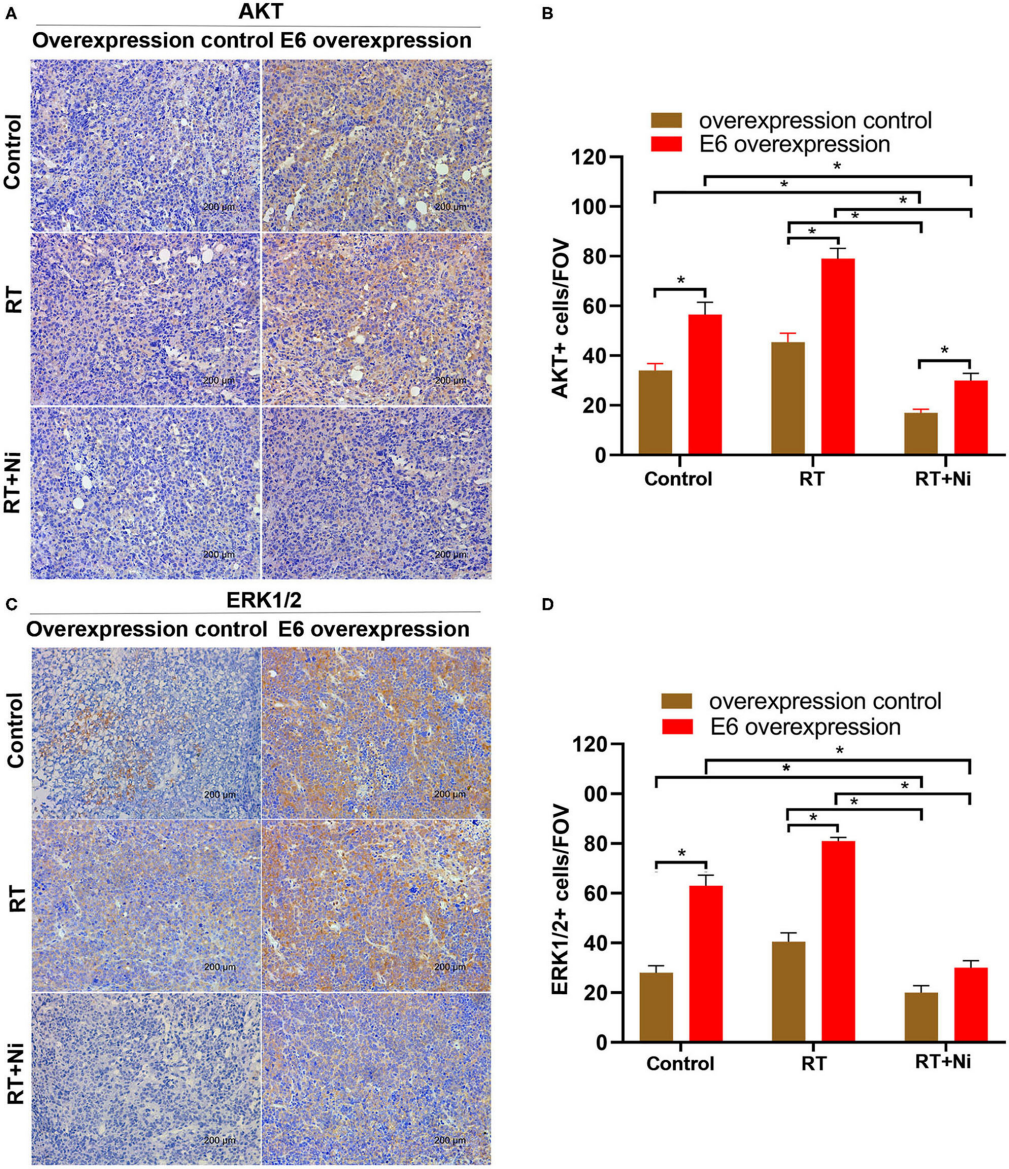
**(A–D)** Expression levels of AKT and ERK1/2 were slightly higher in the RT group compared with the control group, in addition, compared with the control group and the radiotherapy group, radiotherapy combined with nimotuzumab significantly reduced the expression levels of AKT and ERK1/2 (*P < 0.05). Data are representative images (magnification × 200) from each group of tumors.

As shown in Figures 5A,B, in nude xenograft mice, the tumor growth of C33AE6 cells was significantly more rapid than that of C33AT cells from 4 days post-injection onward (*P* < 0.05). Tumor growth was significantly slower in the RT and RT+Ni treatment groups, compared with the control group, from 7 days post-injection onward (*P* < 0.05). Tumor growth in the RT+Ni group was significantly slower than that in the RT group from 7 days post-injection onward (*P* < 0.05), suggesting that nimotuzumab enhanced the tumor regression induced by irradiation. Immunohistochemical analysis revealed the changes of the expression levels of EGFR (Figures 5C,D), AKT (Figures 6A,B), and ERK 1/2 (Figures 6C,D) in the samples with different treatment from the tumor-bearing mice. Compared with the control group, radiotherapy can slightly increase the expression levels of EGFR, AKT, and ERK 1/2 in C33A cells. In addition, compared with the control group and the radiotherapy group, radiotherapy combined with nimotuzumab significantly reduced the expression levels of EGFR, AKT, and ERK 1/2 (*P* < 0.05). Therefore, combined with nimotuzumab significantly inhibited the growth of implanted C33A tumors in mice, which is related to the inhibition of the activation of EGFR-related AKT and ERK 1/2 signaling.

**Figure 7:**
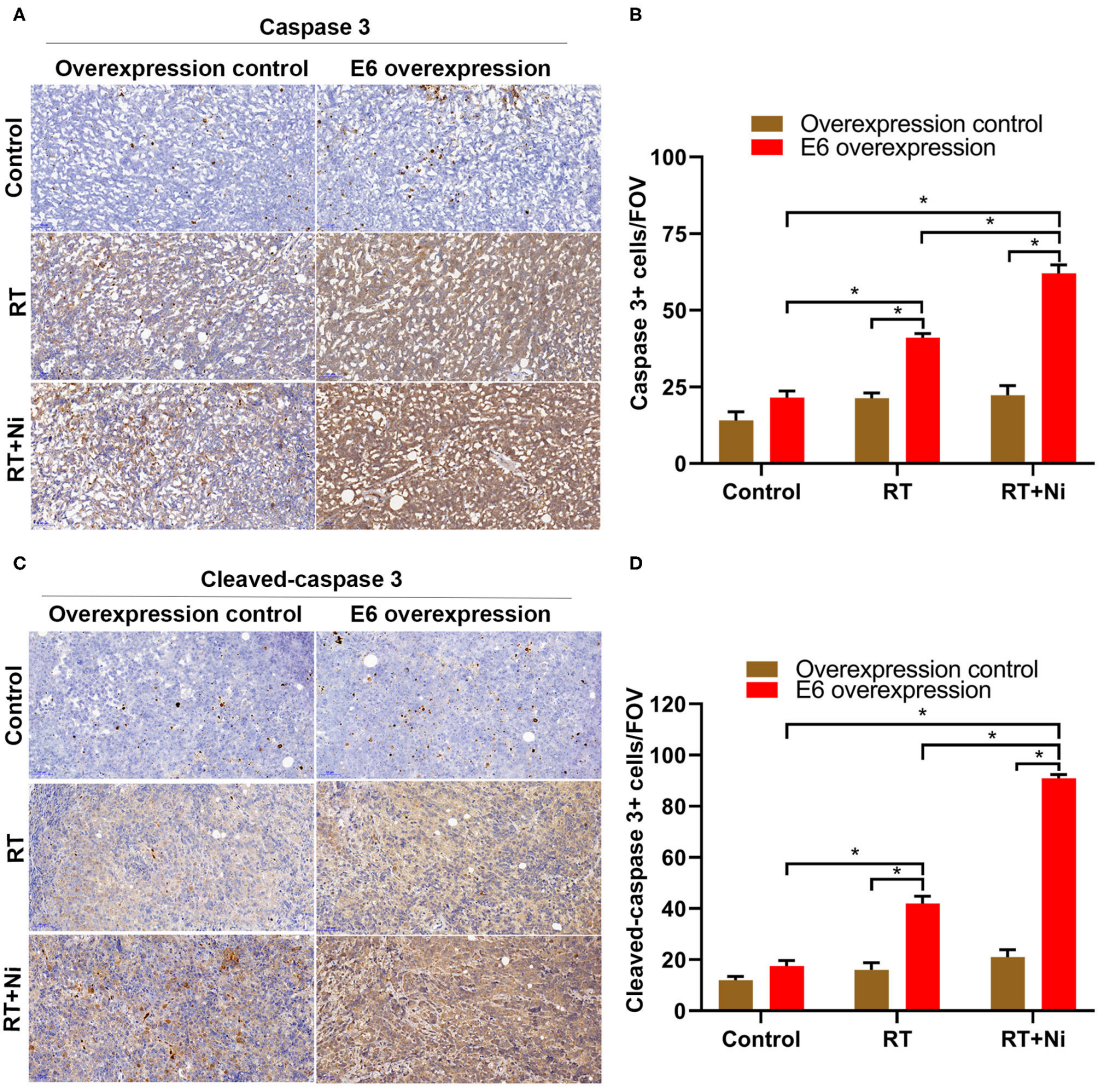
Expression levels of caspase 3 **(A,B)** and cleaved-caspase 3 **(C,D)** were significantly stronger in the RT group and the RT+Ni group, compared with the control group (*P < 0.05). The expression of caspase 3 and cleaved-caspase 3 was also significantly stronger in the RT+Ni group than in the RT group (*P < 0.05). Data are representative images (magnification × 200) from each group of tumors.

As shown in Figure 7, the expression levels of caspase 3 (Figures 7A,B) and cleaved-caspase 3 (Figures 7C,D) were significantly higher in the RT treatment group and the RT+Ni group, compared with the control group (*P* < 0.05). In addition, the expression of caspase 3 and cleaved-caspase 3 in the RT+Ni group was significantly stronger than that in the RT group (*P* < 0.05).

## Discussion

As an HPV-negative and P53-deficient cell line, C33A has been widely used in research on human cervical cancer. In 2010, Hampson et al. transfected HPV E6 and E7 genes into C33A cell lines, respectively. The experimental results showed that C33A cells transfected with E6 grew faster than C33A cells transfected with E7 (24). The authors also conducted *in vitro* and mouse model experiments; the results of these experiments confirmed that when C33A cells were transfected with E6, the tumoricidal effect of ionizing radiation was significantly increased. In contrast, the transfected E7 gene had no significant effect on the radiosensitivity of C33A cells (24). These results suggest that E6 contributed to the enhanced radiosensitivity in a P53-independent way. In support of this assertion, our results also demonstrated that E6-overexpressing C33A cells grew faster and were more sensitive to radiotherapy than control cells, both *in vitro* and *in vivo*.

The interaction between HPV and EGFR has previously been described. HPV infection was previously reported to cause EGFR overexpression in squamous cell carcinoma (25). HPV16 E6 has also been demonstrated to induce EGFR activation in the setting of EGF stimulation. Even in the absence of EGF, E6 was found to increase EGFR phosphorylation, leading to prolonged EGFR, MAPK, and PI3K activation (26). Studies have shown E6 protein expressed by high-risk HPV upregulated EGFR transcription in cervical squamous cell carcinoma by destabilizing the histone demethylase KDM5C on super-enhancers (27). HPV16 E6 is also a target of EGF-induced signaling; its activity was found to be amplified by EGF stimulation in SiHa cervical cancer cells (28). Our results also revealed that expression levels of EGFR, as well as downstream signal molecules AKT and ERK 1/2, were significantly upregulated in C33A cells overexpressing E6. Erlotinib is a small-molecule inhibitor of EGFR that has been shown to slow the growth of locally advanced cervical cancer. In a phase II clinical trial, 36 patients were treated with erlotinib combined with cisplatin and radiotherapy (29). Among this group of patients, 34 (94.4%) achieved a complete response to treatment. The cumulative overall survival rates at 2 and 3 years were 91.7 and 80.6%, respectively; progression-free survival at 2 and 3 years was 80 and 73.8%, respectively. Woodworth et al. (30) also found that erlotinib may prevent immortalization and induce apoptosis in HPV16-positive cervical cancer cells. Collectively, these findings suggest that EGFR inhibitors may improve the efficacy of radiotherapy in cervical cancer.

Nimotuzumab is a humanized IgG1 isotype monoclonal antibody used in the treatment of malignant tumors (18, 19). Because of its ability to bind EGFR, nimotuzumab can competitively interfere with the binding of EGF to EGFR, resulting in the blockade of downstream signaling pathways, inhibiting tumor cell proliferation, promoting tumor cell apoptosis, and enhancing the efficacy of radio- and/or chemotherapy. Although nimotuzumab has been used less frequently in treating cervical cancer, several studies have shown that the combination of nimotuzumab with radio-chemotherapy for locally advanced cervical cancer has a very reliable treatment effect (31–33). Nimotuzumab is therefore a promising option for preoperative neoadjuvant treatment. In an early study by Yang et al. 80 patients with locally advanced cervical cancer were treated with cisplatin or gemcitabine in combination with nimotuzumab, followed by complete pelvic radiotherapy. The results showed that 11 patients had partial relief of symptoms; 59 patients were stable; the rate of disease control reached 88%. Drug side effects and safety were also within acceptable limits (33). In another study, 28 patients with locally advanced cervical cancer received preoperative neoadjuvant therapy. From the 1st week of intensity-modulated radiotherapy (IMRT), nimotuzumab was administered intravenously in combination with cisplatin or nedaplatin, weekly. After 1 month of neoadjuvant treatment, magnetic resonance imaging (MRI) and gynecological evaluations showed a complete clinical response rate of 28.5% and partial response rate of 71.5%. The median survival of the 24 patients who had undergone surgery was 22 months (34). Additional studies have investigated the mechanisms underlying the ability of nimotuzumab to sensitize cancer cells to radiotherapy. Lin et al. found that nimotuzumab significantly enhanced apoptosis in A549 cells irradiated with 2 Gy, compared with A549 cells treated with nimotuzumab or irradiation alone. Combining nimotuzumab with irradiation also significantly increased the number of cells arrested in the G2/M phase, compared with the use of radiotherapy or nimotuzumab treatment alone (35). In another study conducted by Gao et al. PANC-1 pancreatic cancer cells were treated with nimotuzumab, alone, or combined with radiation. The results showed that the inhibition rate, the percentage of G2/M phase arrest, and the rate of apoptosis were all significantly higher in the group treated with nimotuzumab and radiation, compared with the group treated with radiation alone (36).

Caspase 3 (cysteine-aspartate protease 3), known as cysteine protease 3, is a key enzyme for both endogenous and exogenous apoptotic pathways. This enzyme also plays an important role in regulating inflammation, cell proliferation, and cell differentiation. Once activated, caspase 3 causes irreversible cell death by degrading important cellular proteins and activating endonucleases. Caspase 3 is therefore considered to be a marker of apoptosis. However, caspases 3 exists as an inactive zymogen under normal circumstances. When cells undergo apoptosis, caspase 3 is activated to cleaved-caspase 3, which plays a role in promoting apoptosis by degrading low-molecular-weight DNA and causing nuclear translocation. Shinomiya (37) pointed out that radiation-induced apoptosis is a rapid form of apoptotic cell death, related to the activation of caspase 3. Therefore, we sought to detect treatment-induced cancer cell apoptosis in tumor-bearing nude mice with IHC staining against caspase 3 and cleaved-caspase 3. Microscopic observation revealed that most staining for these markers localized to the cytosol. In the present study, the expression levels of caspase 3 and cleaved-caspase 3 were significantly higher in the RT treatment group and the RT+Ni group, compared with the control group, which means that the apoptosis was involved in the radiotherapy and combined therapy, and more activated apoptosis could be observed in the combined therapy.

Aberrant EGF-mediated abnormal signaling plays a vital role in increasing the ability of tumor cells to proliferate and migrate during the growth process. The main downstream signaling pathways for EGFR activation include the Ras mitogen-activated protein kinase (MAPK) cascade, the phosphatidylinositol 3 kinase (PI3K) cascade, and the signal transduction and transcription activator (STAT) cascade (38). We found that radiotherapy alone can slightly increase the expression levels of EGFR and AKT in C33A cells, which may be due to accelerated tumor cell proliferation, anti-apoptosis, and radiation resistance caused by radiation (39). Importantly, nimotuzumab combined with radiotherapy significantly inhibited the activation of EGFR, AKT, and ERK 1/2 in C33A cells. In the GBM (glioblastoma multiforme) model, Nimotuzumab combined with radiotherapy also obtained similar results (40). These data indicate that the inhibitory effect of nimotuzumab on the EGFR signaling enhances the cytotoxic effect of radiation to a certain extent.

In the present study, we found that nimotuzumab combined with radiotherapy in inhibiting E6-promoted C33A cell growth *in vitro* and *in vivo*. This combined effect of nimotuzumab and radiotherapy increased the rates of G2/M cell cycle arrest and apoptosis in E6-overexpressing CSCC *in vitro*. Previous studies showed that, after medication or radiation is applied to tumor cells, cell cycle checkpoints will detect cellular DNA damage and induce in the number a greater proportion of cells remaining in the G2/M phase (41). G2/M arrest has two effects: First, cancer cell mitosis is discontinued, and cell proliferation is inhibited. Second, if cancer cells accumulate and fail to repair DNA damage, apoptosis will be triggered, and G2/M arrest becomes irreversible (41, 42). In addition, our data also shows that nimotuzumab combined with radiotherapy can increase the radiotherapy sensitivity of p53 mutant C33A cells. Therefore, this work broadens our preclinical understanding that EGFR inhibitors can improve radiation anti-tumor and provides an ideal choice for the treatment of p53 mutant cervical squamous cell carcinoma. However, further clinical trials are needed to confirm these findings and assess the potential toxicity of cervical squamous cell carcinoma patients.

## Data Availability Statement

The raw data supporting the conclusions of this article will be made available by the authors, without undue reservation.

## Ethics Statement

The Animal Care and Use Committee at Anhui Medical University approved all experimental procedures, which were designed so as to minimize animal suffering.

## Author Contributions

QZ and YLv conceived and designed research. ZX and HS collected data and wrote the initial paper. QZ conducted research. FZ and WL analyzed and interpreted data. YLi revised the paper. YLi and JC had primary responsibility for final content. All authors read and approved the final manuscript.

## Conflict of Interest

The authors declare that the research was conducted in the absence of any commercial or financial relationships that could be construed as a potential conflict of interest.
